# Heterogeneity in cognitive disability after a major disaster: A natural experiment study

**DOI:** 10.1126/sciadv.abj2610

**Published:** 2021-09-29

**Authors:** Koichiro Shiba, Adel Daoud, Hiroyuki Hikichi, Aki Yazawa, Jun Aida, Katsunori Kondo, Ichiro Kawachi

**Affiliations:** 1 Department of Epidemiology, Harvard T.H. Chan School of Public Health, Boston, MA, USA.; 2 Department of Social and Behavioral Sciences, Harvard T.H. Chan School of Public Health, Boston, MA, USA.; 3 Center for Population and Development Studies, Harvard T.H. Chan School of Public Health, Boston, MA, USA.; 4 Institute for Analytical Sociology, Linköping University, Norrköping, Sweden.; 5 The Division of Data Science and Artificial Intelligence, the Department of Computer Science and Engineering, Chalmers University of Technology, Gothenburg, Sweden.; 6 Division of Public Health, Kitasato University School of Medicine, Kanagawa, Japan.; 7 Department of Oral Health Promotion, Graduate School of Medical and Dental Sciences, Tokyo Medical and Dental University, Tokyo, Japan.; 8 Center for Preventive Medical Sciences, Chiba University, Chiba, Japan.; 9 Center for Gerontology and Social Science, National Center for Geriatrics and Gerontology, Aichi, Japan.

## Abstract

Cognitive disability following traumatic experiences of disaster has been documented; however, little is known about heterogeneity in the association across individuals. In this natural experiment study of approximately 3000 Japanese older adults in an area directly affected by the 2011 Great East Japan Earthquake, the baseline survey was established 7 months before the 2011 earthquake. To inductively identify heterogeneity in postdisaster cognitive disability by predisaster characteristics, we applied a machine learning–based causal inference approach—generalized random forest. We identified strong evidence for heterogeneity in the association between home loss and cognitive disability objectively assessed 2.5 and 5.5 years after the 2011 earthquake. The subgroups with the strongest disaster-dementia associations tended to be from low socioeconomic backgrounds and have predisaster health problems. The study demonstrated that some subpopulations are particularly prone to experience cognitive disability after disasters, which could be overlooked in studies assessing population average associations only.

## Teaser

Older adults with low socioeconomic status and health problems may experience greater postdisaster cognitive disability.

## INTRODUCTION

Disaster-related traumatic experiences have been linked to declines in working memory (*1*, *2*) and exacerbation of dementia among older adults (*3*–*5*). Postulated mechanisms include posttraumatic stress disorder (PTSD) and depression (both risk factors for cognitive disability) (*6*, *7*), decline in social participation, and increased risks of social isolation accompanying residential displacement (*8*). While the existing evidence captures the population average effects of disaster-related stressors on cognitive disability, the adverse impacts of disasters are also likely to vary across individuals. For example, there is considerable evidence suggesting that only a fraction of individuals exposed to traumatic experiences develop subsequent mental health problems, which may lead to heterogeneity in postdisaster cognitive disability too (*9*, *10*). People with previously reported protective factors for postdisaster psychopathology [e.g., no preexisting psychiatric conditions, high socioeconomic status (SES), and social support] may be less likely to experience cognitive disability following disasters too (*11*, *12*). Moreover, living in a community with strong ties (social capital) could mitigate the postdisaster social isolation and prevent cognitive disability among older survivors (*13*, *14*). Studying such heterogeneity will help to identify individuals or groups who are particularly vulnerable (or resilient) to disaster-related stressors. This can, in turn, assist in the allocation of scarce resources and target the delivery of interventions to address the needs of vulnerable subgroups (*15*).

Two methodological challenges have hampered rigorous estimation of heterogeneous associations of disasters with cognitive disability and identification of predisaster characteristics contributing to the heterogeneity. First, rich information on the characteristics of disaster survivors predating disaster onset is rarely available. Studies of disaster survivors typically collect data on these characteristics retrospectively and are therefore subject to recall bias (*16*). Second, most studies assessing heterogeneity rely on a deductive approach, in which the researchers select a limited set of predictors a priori as sources of heterogeneity and statistically test interactions, each variable at a time (*17*). While the deductive approach is useful when investigators have prior knowledge about which factors may modify the disaster-dementia associations (i.e., testing substantive theory), this approach will likely miss other heterogeneity patterns that the investigators do not explicitly search for. However, no study to date has overcome these challenges and examined the post-trauma cognitive disability.

The present study estimated heterogeneous associations of traumatic experiences with subsequent cognitive disability among older adult survivors. We used a recently developed machine learning (ML) algorithm that flexibly and inductively assesses effect heterogeneity—generalized random forest (GRF) (*18*, *19*). Inductive assessment of effect heterogeneity does not require investigators to select specific effect modifiers a priori but rather find them in a data-driven way. We leveraged a unique natural experiment setting stemming from the 2011 Great East Japan Earthquake and Tsunami, wherein a longitudinal cohort study of Japanese older adults established 7 months before the earthquake onset offered an opportunity to use a rich “predisaster” set of data among survivors (*20*).

## RESULTS

Table 1 shows predisaster demographic characteristics of study participants and their cognitive outcomes in the follow-up waves by levels of the disaster damage. Individuals who experienced home loss (versus no home loss or less severe damage) had greater levels of cognitive disability both in 2013 (2.5 years after the onset) and 2016 (5.5 years after). Those who experienced home loss tended to be from lower socioeconomic backgrounds (fewer years of schooling and lower household income) compared to those without home loss, but we did not find such difference in SES for loss of loved ones.

**Table 1.** Cognitive disability outcomes in 2013/2016 and baseline sociodemographic characteristics of analytic samples in 2010 (*n* = 3350). ADL, activity of daily living; and IADL, instrumental activity of daily living.

**Characteristics**	**Overall**	**Home loss**		**Loss of loved ones**	
		**Yes**	**No**	**Yes**	**No**
	**(*n* = 3350)**	**(*n* = 148)**	**(*n* = 3112)**	**(*n* = 1254)**	**(*n* = 2096)**
**Levels of certified** **cognitive disability in** 2013*	0.24 (0.88)	0.57 (1.40)	0.22 (0.85)	0.22 (0.84)	0.25 (0.91)
**Levels of certified** **cognitive disability in** 2016*,**†**	0.37 (1.04)	0.56 (1.21)	0.36 (1.03)	0.34 (0.99)	0.38 (1.07)
**Age, mean (SD)**	73.2 (6.0)	73.6 (6.4)	73.2 (6.0)	72.7 (5.8)	73.5 (6.1)
**Gender, *n* (%)**					
Men	1857 (55%)	87 (59%)	1709 (55%)	723 (58%)	1134 (54%)
Women	1493 (45%)	61 (41%)	1403 (45%)	531 (42%)	962 (46%)
**Marital status, *n* (%)**					
Married	2364 (73%)	98 (74%)	2216 (73%)	889 (73%)	1475 (73%)
Widowed	733 (23%)	30 (23%)	679 (22%)	273 (23%)	460 (23%)
Divorced	83 (2.6%)	2 (1.5%)	77 (2.5%)	29 (2.4%)	54 (2.7%)
Single	39 (1.2%)	0 (0%)	36 (1.2%)	11 (0.9%)	28 (1.4%)
Others	18 (0.6%)	2 (1.5%)	15 (0.5%)	10 (0.8%)	8 (0.4%)
**Living alone, *n* (%)**					
No	2979 (91%)	134 (97%)	2772 (91%)	1141 (93%)	1838 (90%)
Yes	281 (8.6%)	4 (2.9%)	269 (8.8%)	85 (6.9%)	196 (9.6%)
**Education, *n* (%)**					
Less than 6 years	33 (1.0%)	0 (0%)	33 (1.1%)	11 (0.9%)	22 (1.1%)
6–9 years	1103 (34%)	92 (67%)	969 (32%)	467 (38%)	636 (31%)
10–12 years	1417 (44%)	35 (26%)	1346 (44%)	496 (41%)	921 (45%)
13 years or more	676 (21%)	8 (5.8%)	660 (22%)	236 (19%)	440 (22%)
Others	26 (0.8%)	2 (1.5%)	23 (0.8%)	12 (1.0%)	14 (0.7%)
**Job, *n* (%)**					
Working	550 (19%)	23 (20%)	514 (19%)	232 (21%)	318 (17%)
Retired	1892 (64%)	66 (56%)	1782 (64%)	666 (60%)	1226 (66%)
Never worked	520 (18%)	28 (24%)	479 (17%)	211 (19%)	309 (17%)
**Household income,** **mean (SD)^‡^**	231 (141)	170 (127)	234 (141)	227 (142)	233 (140)
**Depressive symptoms,** ***n* (%)^§^**					
Mild/severe depressive symptoms	857 (30%)	45 (37%)	778 (29%)	315 (29%)	542 (30%)
No depressive symptoms	2039 (70%)	77 (63%)	1931 (71%)	783 (71%)	1256 (70%)
**Self-rated health, *n* (%)**					
Very good	417 (13%)	24 (17%)	384 (13%)	159 (13%)	258 (13%)
Good	2336 (71%)	93 (65%)	2183 (71%)	873 (71%)	1463 (71%)
Not good	458 (14%)	15 (11%)	430 (14%)	165 (13%)	293 (14%)
Bad	75 (2.3%)	10 (7.0%)	61 (2.0%)	31 (2.5%)	44 (2.1%)
**Body mass index,** **mean (SD)**	23.6 (3.1)	23.9 (2.8)	23.5 (3.1)	23.6 (3.0)	23.5 (3.1)
**Total IADL, mean (SD)^||^**	11.9 (1.80)	11.4 (2.50)	11.9 (1.76)	12.0 (1.75)	11.8 (1.83)
**ADL, mean (SD)^¶^**	2.98 (0.15)	2.94 (0.31)	2.99 (0.13)	2.99 (0.14)	2.98 (0.15)
**No. of treatment for** **major diseases, mean** **(SD)^#^**	1.47 (1.36)	1.40 (1.28)	1.46 (1.35)	1.46 (1.34)	1.47 (1.37)

*Levels of certified cognitive disability ranged from 0 (no cognitive deficits) to 7 (needs constant treatment in a specialized medical facility) according to the severity of their cognitive disability.

†Calculated among the analytic sample for the cognitive outcome in 2016 (*n* = 2664).

‡Annual household income was divided by the square root of the number of household members to account for household size.

§We used the Geriatric Depression Scale (range: 0 to 15 points; higher scores indicate more depressive symptoms) to assess depressive symptoms.

||IADL was measured by the 13-item Tokyo Metropolitan Institute of Gerontology Index of Competence. Scores ranged from 0 to 13 points, where smaller scores indicate lower functional independence.

¶ADL had three levels (1 = completely needed, 2 = partially needed, and 3 = no help needed).

#We calculated counts of current treatment for major diseases, including cancer, heart diseases, stroke, hypertension, diabetes, obesity, hyperlipidemia, osteoporosis, arthritis, fracture, respiratory diseases, gastrointestinal diseases, liver diseases, psychiatric diseases, dysphagia, visual impairment, hearing loss, dysuria, and insomnia.

**Fig. 1.**
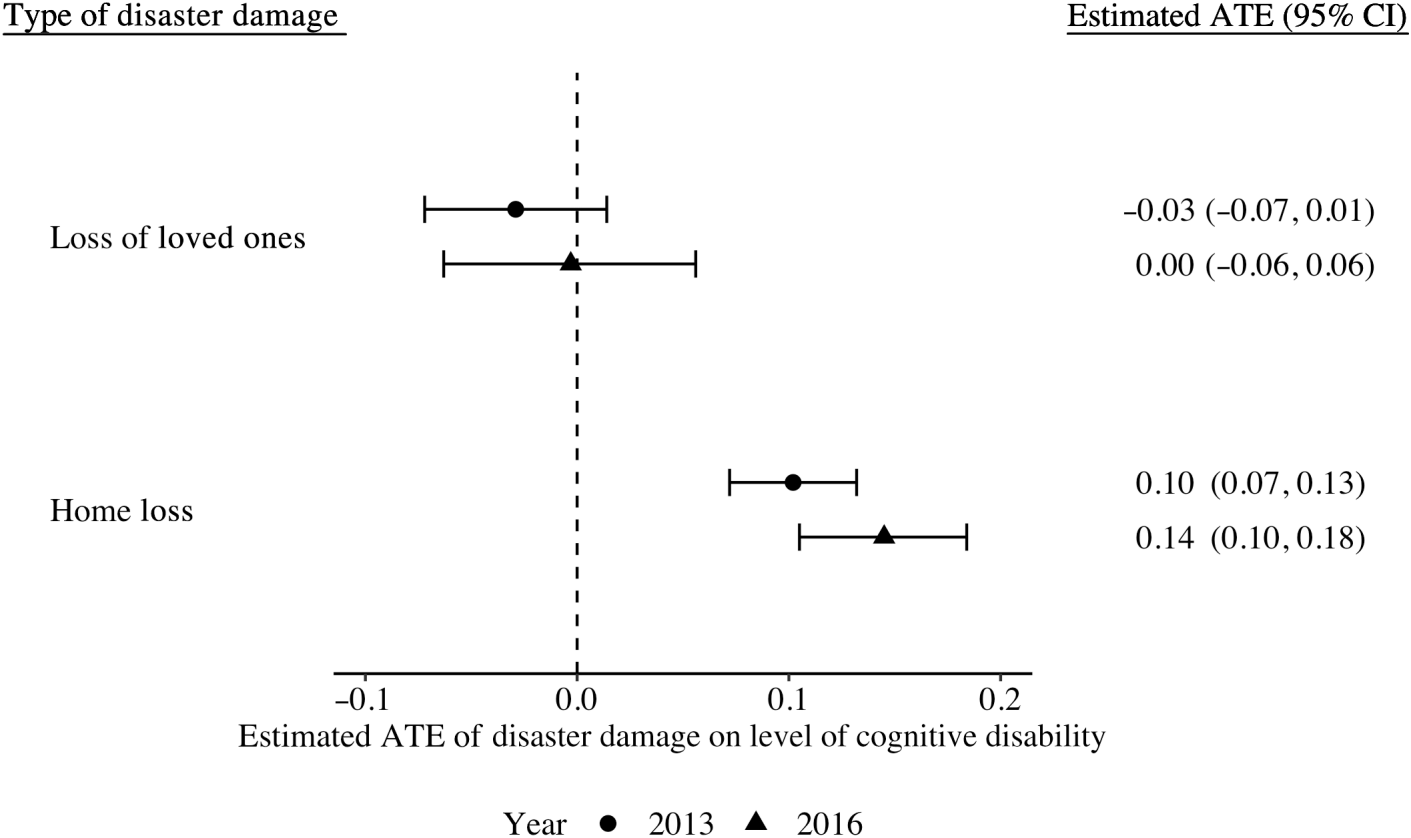
Estimated population ATEs of disaster-related trauma experiences on level of cognitive disability in 2013 and 2016. Population average effects (i.e., ATEs) of the exposures were estimated via the doubly robust targeted maximum likelihood estimation. Models were estimated data adaptively via the SuperLearner using generalized linear models, gradient boosting machine, and neural net as candidate estimators. Levels of certified cognitive disability ranged from 0 (no cognitive deficits) to 7 (needs constant treatment in a specialized medical facility) according to the severity of their cognitive disability. Thus, larger effect estimates indicate greater level of cognitive disability. All models were adjusted for the 51 predisaster demographic and socioeconomic factors, health conditions, psychosocial variables, and behaviors from the 2010 wave.

Figure 1 shows estimated average treatment effects (ATEs) of the disaster damages on cognitive disability. Home loss was associated with increased levels of cognitive disability in 2013 [estimate: 0.10; 95% confidence interval (CI): 0.07, 0.13] and in 2016 (estimate: 0.14; 95% CI: 0.10, 0.18), after adjusting for the 51 predisaster characteristics. There was no strong evidence that loss of loved ones was, on average, associated with cognitive disability (estimate: −0.03; 95% CI: −0.07, 0.01 for 2013 and estimate: −0.03; 95% CI: −0.06, 0.06 for 2016). Sensitivity analysis using different cutoffs for housing damage showed a dose-response relationship that greater damage was associated with greater levels of cognitive disability (fig. S1). Our ad hoc analysis indicated that the diagnosis of stroke in 2010 was likewise associated with subsequent cognitive disability (estimate: 0.19; 95% CI: 0.13, 0.25 for 2013 and estimate: 0.06; 95% CI: −0.01, 0.14 for 2016; fig. S2).

**Fig. 2.**
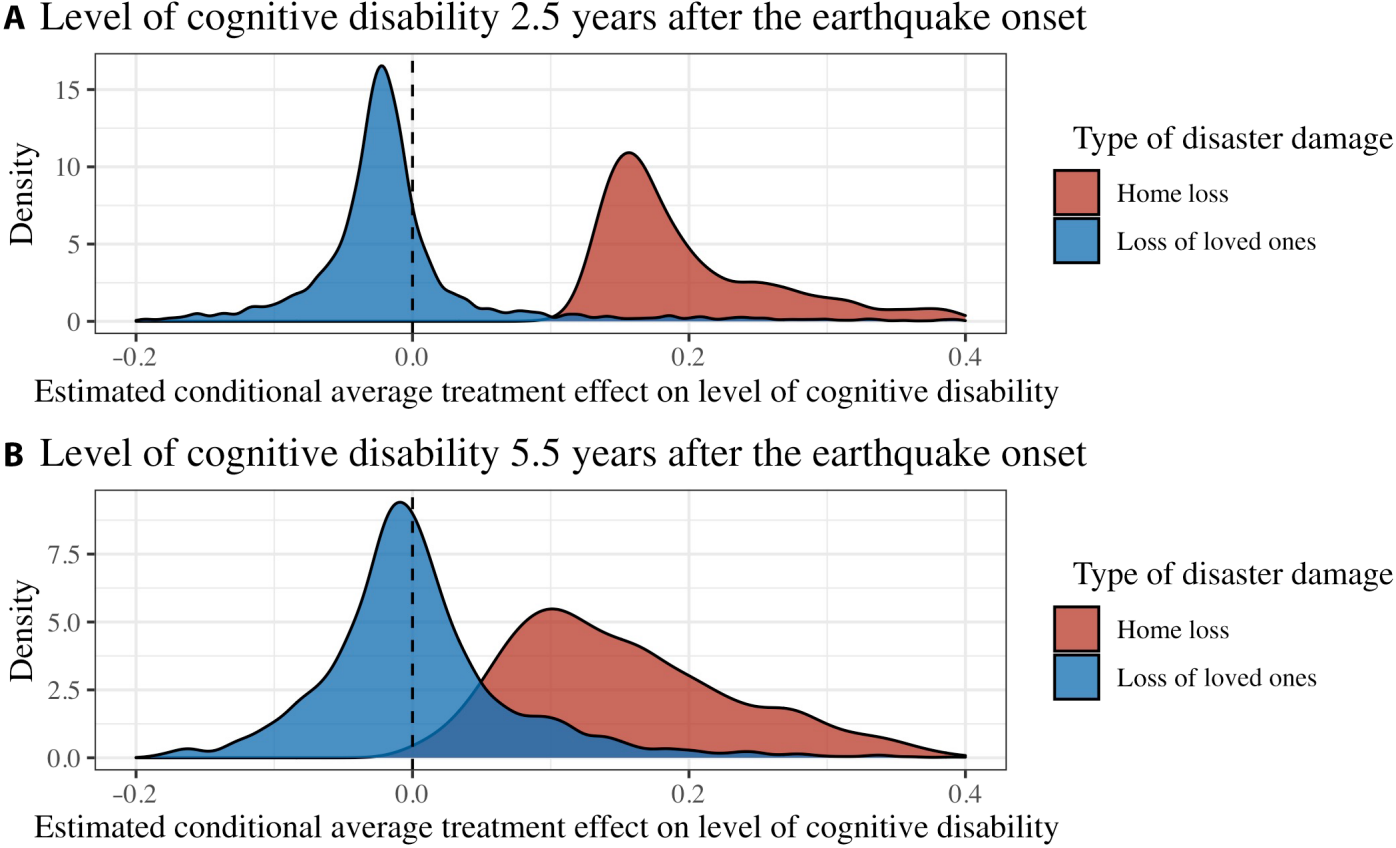
Distributions of estimated CATEs of disaster-related trauma experiences on level of cognitive disability in 2013 and 2016. Heterogeneous effects (i.e., CATEs) were estimated using GRF algorithm, using the 51 predisaster demographic and socioeconomic factors, health conditions, psychosocial variables, and behaviors from the 2010 wave. Levels of certified cognitive disability ranged from 0 (no cognitive deficits) to 7 (needs constant treatment in a specialized medical facility) according to the severity of their cognitive disability. Thus, larger effect estimates indicate greater level of cognitive disability.

Figure 2 (see table S1 for the summary statistics) shows the distributions of the estimated conditional average treatment effects (CATEs)—the effects among subpopulations with identical covariate values—estimated via GRF. Evidence of heterogeneity was strong for home loss (*P* < 0.01 for both 2013 and 2016) and weak for loss of loved ones (*P* = 0.78 for 2013 and *P* = 0.33 for 2016). Despite the heterogeneity, the CATE estimates for home loss showed consistent trends with the ATE estimates and were greater than zero for most individuals (i.e., home loss was associated with cognitive disability for most people; min = 0.09 and max = 1.56 for 2013; min = −0.01 and max = 0.45 in 2016). Sensitivity analysis using different cutoffs for housing damage similarly showed heterogeneity (fig. S3).

Tables 2 and 3 compare the key predisaster characteristics of the resilient group (bottom decile of the CATE distribution) with those of the vulnerable group (top decile) in 2013 and 2016, respectively. Comparisons of all 51 covariates are available in table S2 for the analysis of the outcome in 2013 and table S3 for the analysis of the outcome in 2016. We identified common characteristics of the vulnerable group (i.e., individuals for whom disaster damages were most strongly associated with cognitive disability) for both types of disaster damage and across years. That is, before the disaster onset, the vulnerable group (versus the resilient group) was more likely to be older, not married, living alone, less educated, and not working and had baseline health problems (more depressive symptoms, poor self-rated health, lower functional independence indicated by the Instrumental Activity of Daily Livings and Activity of Daily Living scores, and more treatment of major diseases). Moreover, we found changing patterns with regard to household income. The larger CATE estimates (i.e., vulnerability) for home loss exposure were not associated with income when assessing cognitive disability in 2013 (mean income in 10,000 yen: 212 in the vulnerable versus 227 in the resilient; *P* = 0.4). On the other hand, the vulnerable group for home loss exposure had higher income when we assessed cognitive disability in 2016 (276 versus 187; *P* < 0.001), although the same group was less educated and had baseline health problems (e.g., depressive symptoms).

**Table 2.** Predisaster sociodemographic characteristics of people at the top 10% versus bottom 10% of the estimated CATE of disaster-related trauma experiences on level of cognitive disability in 2013 (*n* = 3350).

**Characteristics**	**Exposure: Home loss**			**Exposure: Loss of loved ones**		
	**Resilient**	**Vulnerable**	*P* value*	**Resilient**	**Vulnerable**	*P* value*
	**(*n* = 335)**	**(*n* = 335)**		**(*n* = 335)**	**(*n* = 335)**	
**CATE estimates,** **mean (SD)^†^**	0.14 (0.01)	0.69 (0.22)	<0.001	−0.11 (0.04)	0.27 (0.22)	<0.001
**Age, mean (SD)**	68.97 (2.97)	81.95 (6.54)	<0.001	76.70 (4.92)	79.80 (7.22)	<0.001
**Gender, *n* (%)**			<0.001			0.13
Men	135 (40%)	202 (60%)		191 (57%)	210 (63%)	
Women	200 (60%)	133 (40%)		144 (43%)	125 (37%)	
**Marital status, *n* (%)**			<0.001			0.046
Married	314 (94%)	167 (50%)		214 (64%)	182 (54%)	
Widowed	16 (4.8%)	154 (46%)		102 (30%)	139 (41%)	
Divorced	3 (0.9%)	5 (1.5%)		8 (2.4%)	7 (2.1%)	
Single	2 (0.6%)	7 (2.1%)		8 (2.4%)	5 (1.5%)	
Others	0 (0%)	2 (0.6%)		3 (0.9%)	2 (0.6%)	
**Living alone, *n* (%)**			<0.001			0.2
No	329 (98%)	304 (91%)		299 (89%)	309 (92%)	
Yes	6 (1.8%)	31 (9.3%)		36 (11%)	26 (7.8%)	
**Education, *n* (%)**			<0.001			<0.001
Less than 6 years	1 (0.3%)	17 (5.1%)		5 (1.5%)	10 (3.0%)	
6–9 years	108 (32%)	192 (57%)		133 (40%)	185 (55%)	
10–12 years	149 (44%)	89 (27%)		130 (39%)	98 (29%)	
13 years or more	72 (21%)	33 (9.9%)		64 (19%)	38 (11%)	
Others	5 (1.5%)	4 (1.2%)		3 (0.9%)	4 (1.2%)	
**Job, *n* (%)**			<0.001			<0.001
Working	105 (31%)	28 (8.4%)		41 (12%)	31 (9.3%)	
Retired	210 (63%)	180 (54%)		219 (65%)	161 (48%)	
Never worked	20 (6.0%)	127 (38%)		75 (22%)	143 (43%)	
**Household income,** **mean (SD)^‡^**	227 (159)	212 (131)	0.4	175 (132)	214 (125)	<0.001
**Depressive** **symptoms,** ***n* (%)^§^**			<0.001			0.3
Mild/severe depressive symptoms	65 (19%)	173 (52%)		147 (44%)	161 (48%)	
No depressive symptoms	270 (81%)	162 (48%)		188 (56%)	174 (52%)	
**Self-rated health,** ***n* (%)**			<0.001			<0.001
Very good	71 (21%)	20 (6.0%)		35 (10%)	17 (5.1%)	
Good	244 (73%)	184 (55%)		220 (66%)	184 (55%)	
Not good	20 (6.0%)	94 (28%)		66 (20%)	111 (33%)	
Bad	0 (0%)	37 (11%)		14 (4.2%)	23 (6.9%)	
**Body mass index,** **mean (SD)**	25.26 (2.39)	23.03 (3.30)	<0.001	23.40 (3.47)	23.46 (3.35)	0.14
**Total IADL, mean** **(SD)^||^**	12.47 (0.80)	8.96 (3.19)	<0.001	12.07 (1.05)	8.57 (3.11)	<0.001
**ADL, mean (SD)^¶^**	3.00 (0.00)	2.88 (0.38)	<0.001	2.99 (0.12)	2.91 (0.36)	<0.001
**No. of treatment for** **major diseases,** ***n* (%)^#^**	1.17 (1.08)	2.04 (1.69)	<0.001	1.96 (1.70)	1.90 (1.65)	0.7

*P values for between-group differences. We used Wilcoxon rank sum test for continuous variables and Fisher’s exact test for categorical variables.

†Heterogeneous effects (i.e., CATEs) were estimated via the GRF algorithm, using the 51 predisaster demographic and socioeconomic factors, health conditions, psychosocial variables, and behaviors from the 2010 wave. Levels of certified cognitive disability ranged from 0 (no cognitive deficits) to 7 (needs constant treatment in a specialized medical facility) according to the severity of their cognitive disability. Bottom 10% of the CATE distributions were labeled as a Resilient group, because they showed weaker associations between disaster damage and cognitive disability. Top 10% of the CATE distributions were labeled as a Vulnerable group, because they showed stronger associations between disaster damage and cognitive disability.

‡Annual household income was divided by the square root of the number of household members to account for household size.

§We used the Geriatric Depression Scale (range: 0 to 15 points; higher scores indicate more depressive symptoms) to assess depressive symptoms.

||IADL was measured by the 13-item Tokyo Metropolitan Institute of Gerontology Index of Competence. Scores ranged from 0 to 13 points, where smaller scores indicate lower functional independence.

¶ADL had three levels (1 = completely needed, 2 = partially needed, and 3 = no help needed).

#We calculated counts of current treatment for major diseases, including cancer, heart diseases, stroke, hypertension, diabetes, obesity, hyperlipidemia, osteoporosis, arthritis, fracture, respiratory diseases, gastrointestinal diseases, liver diseases, psychiatric diseases, dysphagia, visual impairment, hearing loss, dysuria, and insomnia.

**Table 3.** Predisaster sociodemographic characteristics of people at the bottom 10% versus top 10% of the estimated CATE of disaster-related trauma experiences on level of cognitive disability in 2016 (*n* = 2664).

**Characteristics**	**Exposure: Home loss**			**Exposure: Loss of loved ones**		
	**Resilient**	**Vulnerable**	*P* value^*^	**Resilient**	**Vulnerable**	*P* value^*^
	**(*n* = 267)**	**(*n* = 267)**		**(*n* = 267)**	**(*n* = 267)**	
**CATE Estimates,** **mean (SD)^†^**	0.03 (0.02)	0.34 (0.04)	<0.001	−0.11 (0.03)	0.16 (0.07)	<0.001
**Age, mean (SD)**	68.55 (2.66)	80.45 (3.15)	<0.001	78.79 (5.16)	74.17 (6.00)	<0.001
**Gender, *n* (%)**			0.027			0.1
Men	172 (64%)	147 (55%)		145 (54%)	164 (61%)	
Women	95 (36%)	120 (45%)		122 (46%)	103 (39%)	
**Marital status, *n* (%)**			<0.001			0.03
Married	221 (83%)	171 (64%)		192 (72%)	165 (62%)	
Widowed	33 (12%)	83 (31%)		67 (25%)	83 (31%)	
Divorced	9 (3.4%)	6 (2.2%)		4 (1.5%)	10 (3.7%)	
Single	2 (0.7%)	6 (2.2%)		4 (1.5%)	5 (1.9%)	
Others	2 (0.7%)	1 (0.4%)		0 (0%)	4 (1.5%)	
**Living alone, *n* (%)**			0.093			<0.001
No	249 (93%)	238 (89%)		254 (95%)	230 (86%)	
Yes	18 (6.7%)	29 (11%)		13 (4.9%)	37 (14%)	
**Education, *n* (%)**			0.004			<0.001
Less than 6 years	1 (0.4%)	5 (1.9%)		3 (1.1%)	6 (2.2%)	
6–9 years	72 (27%)	95 (36%)		92 (34%)	138 (52%)	
10–12 years	145 (54%)	105 (39%)		106 (40%)	90 (34%)	
13 years or more	48 (18%)	59 (22%)		63 (24%)	29 (11%)	
Others	1 (0.4%)	3 (1.1%)		3 (1.1%)	4 (1.5%)	
**Job, *n* (%)**			<0.001			0.008
Working	68 (25%)	15 (5.6%)		30 (11%)	33 (12%)	
Retired	156 (58%)	198 (74%)		186 (70%)	154 (58%)	
Never worked	43 (16%)	54 (20%)		51 (19%)	80 (30%)	
**Household income,** **mean (SD)^‡^**	187 (144)	276 (115)	<0.001	220 (143)	181 (108)	<0.001
**Depressive** **symptoms,** ***n* (%)^§^**			<0.001			<0.001
Mild/severe depressive symptoms	34 (13%)	81 (30%)		22 (8.2%)	206 (77%)	
No depressive symptoms	233 (87%)	186 (70%)		245 (92%)	61 (23%)	
**Self-rated health,** ***n* (%)**			<0.001			<0.001
Very good	38 (14%)	27 (10%)		39 (15%)	7 (2.6%)	
Good	211 (79%)	191 (72%)		203 (76%)	134 (50%)	
Not good	17 (6.4%)	40 (15%)		23 (8.6%)	104 (39%)	
Bad	1 (0.4%)	9 (3.4%)		2 (0.7%)	22 (8.2%)	
**Body mass index,** **mean (SD)**	24.69 (3.47)	22.80 (2.46)	<0.001	22.39 (2.50)	23.76 (3.45)	<0.001
**Total IADL, mean** **(SD)^||^**	12.03 (1.46)	12.17 (1.50)	0.053	12.38 (0.81)	9.23 (2.58)	<0.001
**ADL, mean (SD)^¶^**	3.00 (0.00)	3.00 (0.06)	0.3	3.00 (0.00)	2.96 (0.23)	0.004
**No. of treatment for** **major diseases,** ***n* (%)^#^**	1.21 (1.14)	1.77 (1.51)	<0.001	1.62 (1.40)	1.75 (1.63)	0.6

*P values for between-group differences. We used Wilcoxon rank sum test for continuous variables and Fisher’s exact test for categorical variables.

†Heterogeneous effects (i.e., CATEs) were estimated via the GRF algorithm, using the 51 predisaster demographic and socioeconomic factors, health conditions, psychosocial variables, and behaviors from the 2010 wave. Levels of certified cognitive disability ranged from 0 (no cognitive deficits) to 7 (needs constant treatment in a specialized medical facility) according to the severity of their cognitive disability. Bottom 10% of the CATE distributions were labeled as a Resilient group, because they showed weaker associations between disaster damage and cognitive disability. Top 10% of the CATE distributions were labeled as a Vulnerable group, because they showed stronger associations between disaster damage and cognitive disability.

‡Annual household income was divided by the square root of the number of household members to account for household size.

§We used the Geriatric Depression Scale (range: 0 to 15 points; higher scores indicate more depressive symptoms) to assess depressive symptoms.

||IADL was measured by the 13-item Tokyo Metropolitan Institute of Gerontology Index of Competence. Scores ranged from 0 to 13 points, where smaller scores indicate lower functional independence.

¶ADL had three levels (1 = completely needed, 2 = partially needed, and 3 = no help needed).

#We calculated counts of current treatment for major diseases, including cancer, heart diseases, stroke, hypertension, diabetes, obesity, hyperlipidemia, osteoporosis, arthritis, fracture, respiratory diseases, gastrointestinal diseases, liver diseases, psychiatric diseases, dysphagia, visual impairment, hearing loss, dysuria, and insomnia.

## DISCUSSION

This prospective study of older survivors from the 2011 Great East Japan Earthquake examined the heterogeneous associations of disaster-related traumatic experiences and subsequent cognitive disability. There are three main findings. First, we found strong evidence of the population average associations with cognitive disability 2.5 and 5.5 years after the disaster onset for home loss exposure but not for loss of loved ones. Second, we identified large heterogeneity in individual responses to disaster damages (represented by the widespread distributions for the estimated CATEs), especially for home loss exposure. Third, we inductively identified patterns in predisaster characteristics of individuals who were most susceptible to the postdisaster cognitive disability.

Our finding for the population average associations of home loss with cognitive disability and the null results for loss of loved ones in this study are consistent with existing literature (*1*, *4*, *5*, *21*). The effect sizes for home loss (estimate: 0.10; 95% CI: 0.07, 0.13 for 2013 and estimate: 0.14; 95% CI: 0.10, 0.18 for 2016) are clinically important. Our ad hoc analysis showed that the effects of home loss on subsequent cognitive disability may be comparable with diagnosis of stroke, a well-established risk factor of cognitive disability, in 2010 (*22*). We also found considerable effect heterogeneity for home loss. We demonstrated that there were subpopulations for whom experiences of home loss led to substantially greater cognitive disability (mean CATEs in the most vulnerable group = 0.69 for 2013 and 0.34 for 2016). Examining only average relationships masks this heterogeneity and overlook particularly vulnerable subgroups.

Predisaster characteristics of vulnerable individuals—those for whom the home loss exposure was estimated to be more detrimental—were mostly consistent with the existing evidence from deductive tests of heterogeneity in the trauma research literature (*11*, *12*, *23*). The vulnerable individuals tended to be older, not married, living alone, less educated, and not working and had baseline health problems. These findings support our hypothesis that heterogeneity in cognitive disability following disasters arises from the differential likelihood of experiencing postdisaster (i) mental health problems and (ii) social isolation across individuals (*6*, *24*). For example, predisaster depression, a known risk factor of PTSD, was also more prevalent among the vulnerable group in this study (e.g., 52% versus 19% in the resilient group in 2013 in Table 2) (*11*). Factors such as old age, nonmarried status, and living alone might have accelerated social isolation among the exposed individuals (*25*). In addition to the preventive efforts directly targeting cognitive disability, interventions targeting these intermediate mechanisms may further prevent postdisaster cognitive disability. Such an intervention includes allocating resources to build community centers inside the temporary settlements to facilitate social participation and cognitive resilience, because those with lower socioeconomic backgrounds tend to be relocated to temporary settlements after home loss rather than rebuilding new homes (*26*). An example is “ibasho cafes”—an initiative developed in the aftermath of the Great East Japan Earthquake of 2011, which included an elder-created and managed community hub, a café, a vegetable garden, a farmers’ market, a ramen noodle shop, a daycare, an evacuation center, and a community resource center in which elders teach cultural traditions to younger people (*27*).

We also obtained an additional insight that the deductive approach could have missed. The most vulnerable group for the cognitive disability in 2016 was characterized not only by lower educational attainment and more depressive symptoms before the disaster but also by higher household income (as shown in Table 3). In prior work examining heterogeneity deductively, higher SES such as higher income alone is typically linked to resilience to trauma (*16*). By contrast, our inductive approach for effect heterogeneity allowed for complex interactions between multiple characteristics. For example, our analysis suggested that higher income when coupled with low education could result in greater vulnerability. Some prior evidence suggests that such individuals with status inconsistency (e.g., discordance between educational attainment versus earned income) may be at increased risk of engaging in high risk behaviors, such as excess drinking, which may contribute to cognitive disability (*28*). Although the mechanisms for our unexpected finding remain unclear, future research is warranted to understand the complex heterogeneity.

Our study has three strengths. First, we leveraged natural experiment data, with a rich set of information about survivors collected before the disaster exposure. Second, we applied a ML-based causal inference approach to study effect heterogeneity. This method allowed us to identify complex sources of heterogeneity that the common deductive approach for effect heterogeneity might have missed. Third, the cognitive disability outcome data were obtained from the record linkage to the Japanese long-term care insurance (LTCI) data that objectively assessed the severity of cognitive disability during home visits.

Four limitations should be noted. First, our ATE and CATE estimates are, as is the case with any observational study, based on the assumption that the 51 covariates that we included in GRF sufficed for causal identification. Although the distributions of disaster-related traumatic experiences are likely endogenous and we cannot rule out the possibility of unmeasured confounders (e.g., SES in early life), we conducted rigorous adjustment of the survivors’ predisaster characteristics by leveraging our natural experimental design (disaster damages occurred during the follow-up of the preexisting cohort study), thereby minimizing the magnitude of residual bias by an unmeasured confounder (*29*). Second, the current findings do not tell us which characteristics we can intervene upon to mitigate the effects of disaster damages because we chose the covariates to adjust for confounding between exposure to disaster stressors and cognitive disability, but they do not necessarily suffice to adjust for confounding between each predisaster characteristics and the cognitive disability (*30*). Instead, the results predict which subpopulations are at exceptionally high risk of postdisaster cognitive disability. Our approach may be useful for targeting the delivery of interventions to preserve cognitive health. Although the information on some predisaster characteristics we assessed (e.g., social support) is typically unavailable in nonresearch settings, our findings also revealed characteristics of vulnerable individuals (e.g., SES and preexisting health problems) that can be identified using administrative data and clinical records. Third, our exposure assessment was relatively crude and may have overlooked additional variation across individuals. For example, the effects of home loss may differ depending on its value, but we did not have such information. For example, the difference in the amount of wealth lost due to home loss may partly explain why the vulnerable groups for the CATEs of home loss on cognitive disability in 2016 tended to have higher income. Fourth, selection bias is possible, because sample attrition due to loss to follow-up may have been associated with the disaster-related traumatic exposures and shared prior common causes with the outcome (e.g., prior mental health problems) (*31*). The resulting selection bias from such loss to follow-up is likely to underestimate the true causal effects of the disaster exposures (*32*).

In conclusion, our natural experiment study demonstrated considerable heterogeneity in home loss’ adverse impacts from the 2011 Great East Japan Earthquake on cognitive disability among older survivors. Our findings identified subpopulations for whom the same traumatic experience may be particularly toxic, which would be overlooked had we estimated only the population average effects. We also demonstrated that the inductive estimation of effect heterogeneity based on the ML technique allows complex interactions between characteristics and identifies heterogeneity that the conventional deductive approach can miss. Assessing such heterogeneity can contribute to more targeted postdisaster public health interventions to maintain survivors’ cognitive health.

## MATERIALS AND METHODS

### Data

We used data from the Iwanuma Study, which is part of a nationwide cohort study of Japanese older adults [the Japan Gerontological Evaluation Study (JAGES)] (*33*). Iwanuma city was one of the field sites of the JAGES located in Miyagi Prefecture (population, 44,187 in 2010), approximately 80 km (128 miles) from the epicenter of the 2011 Great East Japan Earthquake. The baseline survey of the Iwanuma Study was conducted in August 2010, 7 months before the disaster onset. JAGES conducted a census of all residents ≥65 years old in Iwanuma city (*n* = 8576) and obtained responses from 4957 residents (response rate = 57.8%).

**Fig. 3.**
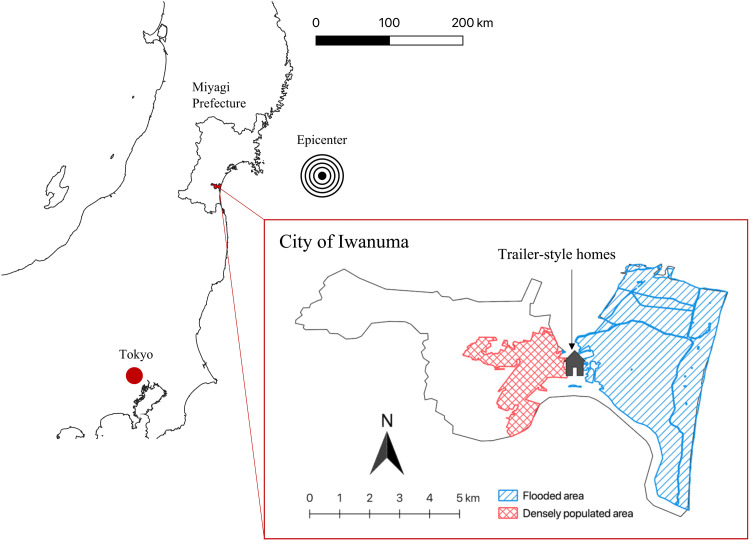
Map of Iwanuma City.

The Great East Japan Earthquake (the Richter scale: 9.0) occurred on 11 March 2011. The earthquake and the subsequent tsunami caused devastating damage to the city of Iwanuma, killing 180 residents, damaging 5542 houses, and inundating 48% of the land area in Iwanuma (Fig. 3) (*34*).

**Fig. 4.**
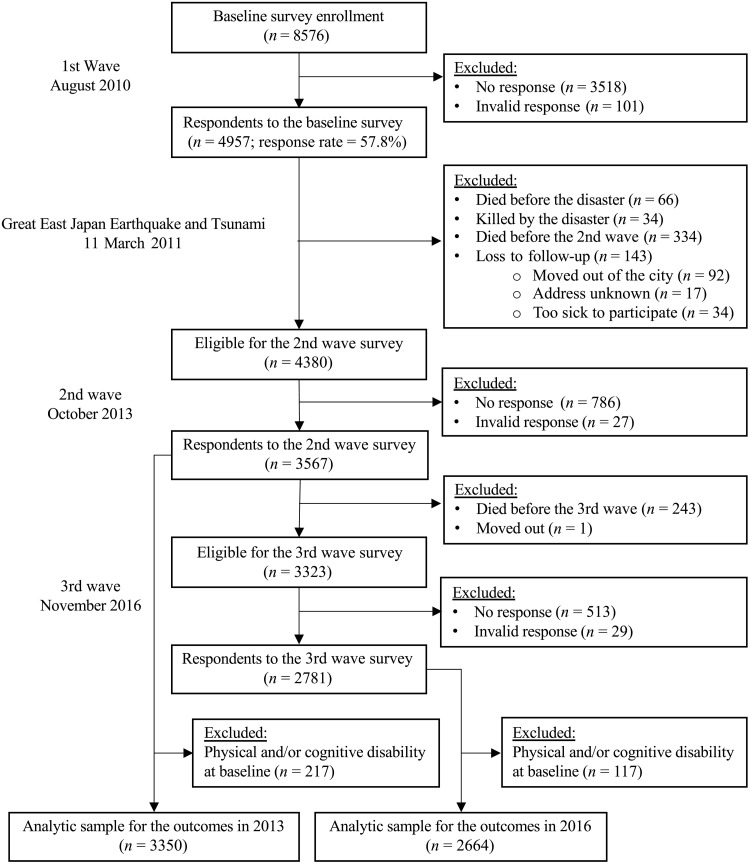
Sample flow chart.

There were two follow-up surveys targeting the baseline respondents who survived the disaster. The first follow-up survey was conducted in October 2013, approximately 2.5 years after the disaster. Of the eligible survivors (*n* = 4380), we obtained valid responses from 3567 subjects (follow-up rate = 81.4%). In November 2016, approximately 5.5 years after the disaster, JAGES conducted the second follow-up survey and recontacted all previous wave respondents. Of the 3323 eligible study participants, we obtained valid responses from 2781 subjects (follow-up rate = 60.8%). To address potential reverse causation, we excluded those who had physical or cognitive disability at baseline. Thus, any change in levels of cognitive disability among this study population is a new onset of cognitive disability. We obtained the final analytic samples (*n* = 3350 for the analysis of the outcome in 2013 and *n* = 2664 for the analysis of the outcome in 2016). Figure 4 summarizes the flow of study participant selection.

### Measurement

#### Outcome

Our outcome of interest was the level of cognitive disability in 2013 and 2016 measured by a standardized in-home assessment under the Japanese LTCI scheme established in 2000 (*35*). All study participants (≥65 years old) were registered for the national LTCI, because it is mandatory for everyone ≥40 years old in Japan. Every applicant who requested to receive long-term care services was assessed for eligibility by a trained investigator dispatched to their homes. Following the assessment, the applicant’s level of cognitive disability was classified into one of seven levels with increasing severity (1: “suffering some cognitive deficits but otherwise almost completely independent” to 7: “needs constant treatment in a specialized medical facility”; see table S4 for the detailed outcome definitions and table S5 for the distributions of each level of disability in the analytic samples). In a prior validation study, level 1 on the seven-point scale has been shown to be correlated with a 0.5-point rating on the Clinical Dementia Scale (specificity and sensitivity = 0.88, respectively) (*36*). The in-home assessment of cognitive disability was also shown to be correlated with the Mini-Mental State Examination (Spearman’s correlation = −0.73, *P* < 0.01) and independent physician’s assessment (Pearson’s correlation = 0.80, *P* < 0.01) (*37*, *38*).

On the basis of this information, we defined a continuous outcome variable for cognitive disability ranging from 0 to 7. Individuals assessed as having no cognitive deficit and those who did not apply for the care services (i.e., people not needing long-term care services) received the value of zero. We obtained the cognitive disability information in 2013 and 2016 for each individual from their initial assessment or subsequent annual reassessments. The same rating scale was used to assess cognitive disability in 2010, and those with baseline cognitive disability of level 1 or greater were excluded from the analysis.

#### Exposure

In the 2013 follow-up survey, participants retrospectively reported two types of traumatic events stemming from the disaster (hereafter, “disaster damage”): home loss and loss of loved ones. Housing damage in Iwanuma was externally assessed by property inspectors and classified into five levels: (i) no damage, (ii) partial, (iii) minor, (iv) major, and (v) complete destruction (*39*). Criteria for each level of housing damage are available in table S6. We created a binary variable representing home loss (1 = “complete destruction” and 0 = “no damage/less severe damage”), because previous evidence has documented that complete home loss was a unique predictor of deteriorated health outcomes after the disaster (*40*, *41*). Respondents also reported loss of loved ones (close friends and/or relatives; 1 = yes, 0 = no).

#### Covariates

We selected 51 predisaster factors from the baseline (2010) survey wave, including four demographic characteristics (gender, age, marital status, and living alone), three measures of SES (educational attainment, employment status, and equivalized household income), 24 health conditions, 14 psychosocial factors, and six behavioral factors (see table S7 for the complete list of the selected variables). We chose these factors because they were likely to operate as confounders (i.e., predictors of cognitive disability distributed differently across the levels of the disaster damages), effect modifiers (i.e., associations between the disaster damages and cognitive disability differ by the levels of these factors), or both.

### Statistical analysis

We conducted the following analyses. First, we estimated the population average associations between the disaster damages and cognitive disability in 2013 and 2016 (also known as ATEs). ATEs quantify the difference in the mean levels of cognitive disability had everyone in the population (i.e., older adults in Iwanuma) been exposed to the damages versus had nobody been exposed, *E*[*Y*_*a*=1_ − *Y*_*a*=0_]. In estimating ATEs, we used doubly robust targeted maximum likelihood estimation (TMLE) (*42*). This approach estimates both the exposure (propensity) model and outcome model and yields unbiased estimates for the ATEs if either of the two models is consistently estimated. Hence, the approach is more robust to model misspecification. We conducted even more robust and stable estimation by fitting both exposure and outcome models data adaptively via the SuperLearner, an ensemble method that uses weighted combinations of multiple ML algorithms (*43*). For the set of SuperLearner algorithms, we used generalized linear models, gradient boosting machine, and neural net as candidate estimators (*44*, *45*). TMLE and Super Learning were performed using the ltmle and SuperLearner R packages (*46*, *47*).

Second, to examine heterogeneous associations, we estimated CATEs of the disaster damages on cognitive disabilitys. Formally, CATE is the effect of an exposure conditional on a set of covariates

$$E[Y_{a=1}-Y_{a=0}|L]$$

where *Y_a_* is the potential outcome *Y* under the binary treatment *A* = *a*, and ***L*** is a set of covariates (confounders and/or effect modifiers). We applied a ML approach called the GRF—an algorithm that adapts the family of random forest (RF) estimators for efficient nonparametric estimation of causal effects—to estimate CATEs of the disaster damages on cognitive disability (*18*, *48*). RF models learn ensembles of regression or classification trees, each tree fitting a different resampled population and covariate set, to estimate and reduce model variance. Each tree learns a set of rules (e.g., age ≥75 versus <75), which partition the population of units into different leaves of the tree. The predicted outcome for a new unit is the average of outcomes for observed units assigned to the same leaf; the prediction of the forest is the average of the predictions of all trees. GRF targets and assesses the contrast in the average outcome between the treated versus untreated individuals in each leaf (i.e., CATEs) rather than predicting the average outcome itself. Estimating CATEs via GRF is advantageous because it does not suffer from the model specification assumptions of a deductively and parametrically specified statistical model, which can be biased and potentially miss complex patterns in heterogeneity. We performed GRF for each of the four exposure-outcome combinations (two types of disaster damages*outcomes from two time points) and estimated CATE for each individual in the sample to assess the distributions of the CATEs using the R package “grf” (*49*). We statistically tested for the presence of heterogeneity by comparing estimated ATEs for the groups above and below the median CATE estimate, following the recommendation by the authors of the original GRF article (*19*).

Third, to assess the most salient heterogeneity, we compared characteristics of the top 10% and the bottom 10% of the CATE distributions. The top 10% (i.e., those with the greatest level of cognitive disability following disaster damage) was labeled as a “Vulnerable” group, and the bottom 10% (i.e., those with the lowest level of cognitive disability following disaster damage) was labeled as “Resilient” group. Last, as a sensitivity analysis, we examined whether results were robust to using different cutoffs to create binary housing damage variables. We performed imputation of missing data using random forest via the R package “missforest” (*50*). All analyses were performed using R, version 3.6.0 (R Foundation for Statistical Computing, Vienna, Austria).
