# Supplementary material for: Heterogeneity in cognitive disability after a major disaster: A natural experiment study
Source: Sci Adv. 2021 Sep 29;7(40):eabj2610. doi: 10.1126/sciadv.abj2610 (PMC8480922; doi:10.1126/sciadv.abj2610)
Supplement: Supplementary file 1 — Figs. S1 to S3 Tables S1 to S7 [file sciadv.abj2610_sm.pdf]

Science Advances
AAAS

Supplementary Materials for

Heterogeneity in cognitive disability after a major disaster: A natural experiment study

Koichiro Shiba*, Adel Daoud, Hiroyuki Hikichi, Aki Yazawa, Jun Aida, Katsunori Kondo, Ichiro Kawachi

*Corresponding author. Email: shiba_k@g.harvard.edu

Published 29 September 2021, *Sci. Adv.* **7**, eabj2610 (2021)
DOI: 10.1126/sciadv.abj2610

This PDF file includes:

Figs. S1 to S3
Tables S1 to S7

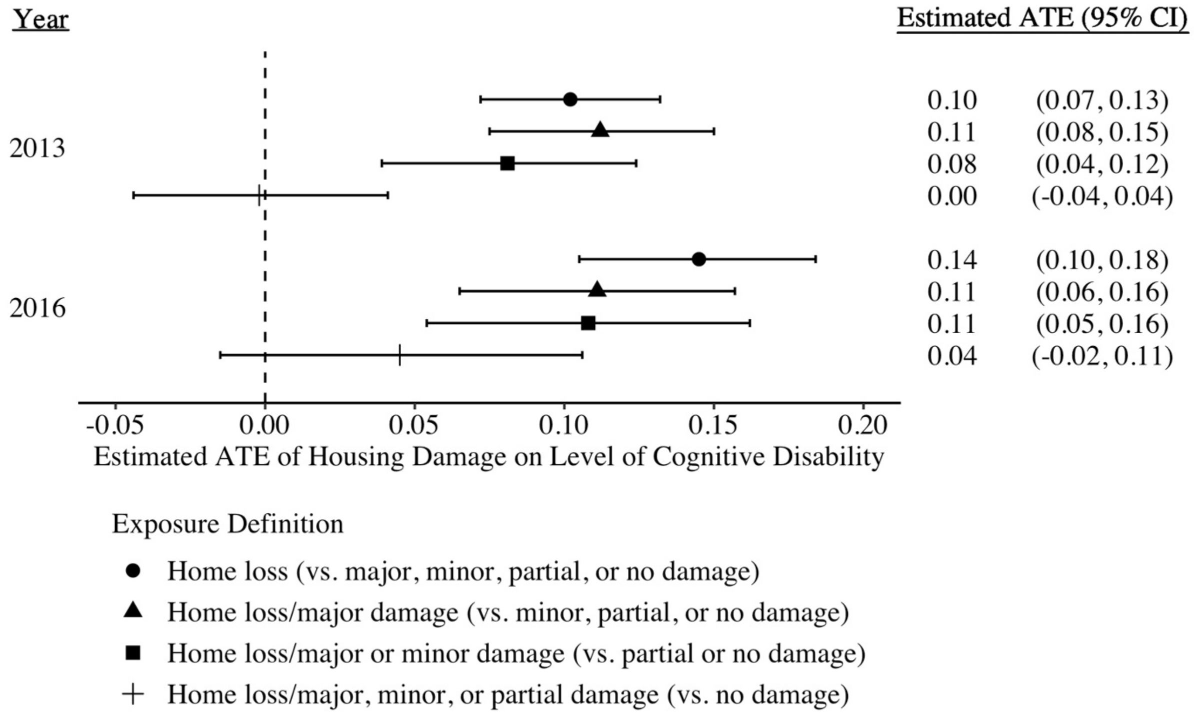

Figure S1. Sensitivity Analysis for Population Average Treatment Effects of Housing Damage on Level of Cognitive Disability in 2013 and 2016.

Population average effects (i.e., average treatment effects) of the exposures were estimated via the doubly-robust targeted maximum likelihood estimation. Models were estimated data-adaptively via the SuperLearner using generalized linear models, gradient boosting machine, and neural net as candidate estimators. Levels of certified cognitive disability ranged from 0 (no cognitive deficits) to 7 (needs constant treatment in a specialized medical facility) according to the severity of their cognitive disability. Thus, larger effect estimates indicate greater level of cognitive disability. All models were adjusted for the 51 pre-disaster demographic and socioeconomic factors, health conditions, psychosocial variables, and behaviors from the 2010 wave.

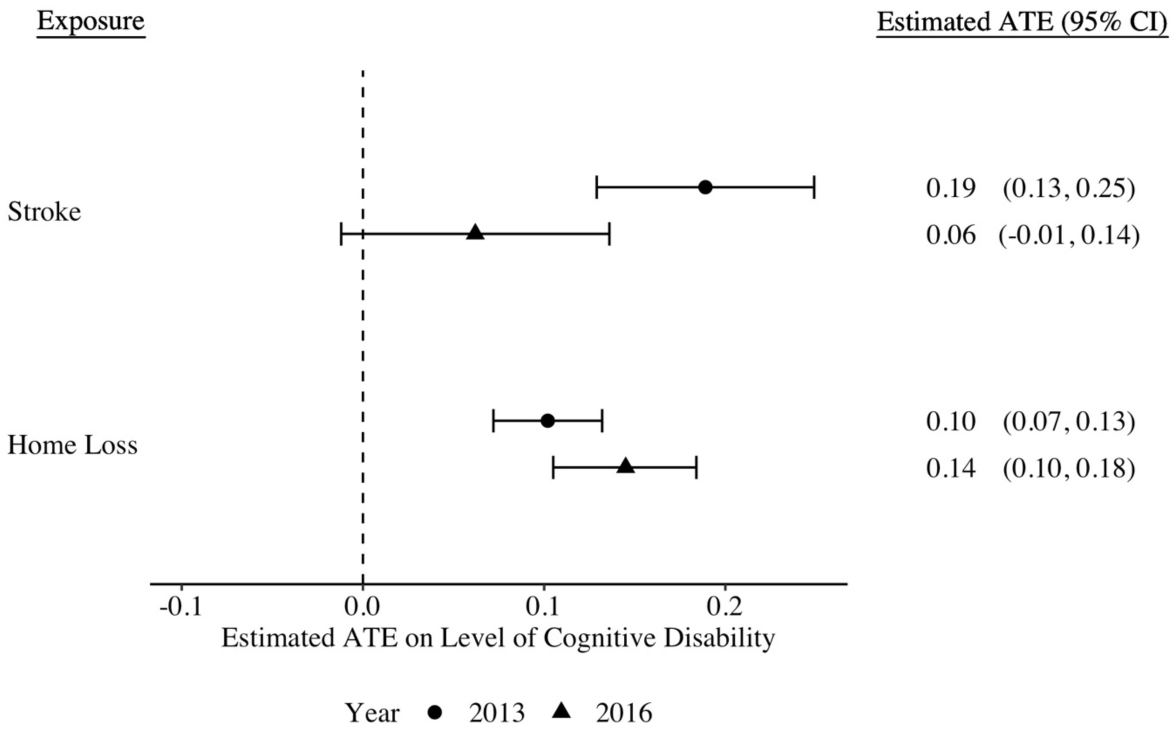

Figure S2. Estimated Population Average Treatment Effects of Home and Baseline Diagnosed Stroke on Level of Cognitive Disability in 2013 and 2016.

Population average effects (i.e., average treatment effects) of the exposures were estimated via the doubly-robust targeted maximum likelihood estimation. Models were estimated data-adaptively via the SuperLearner using generalized linear models, gradient boosting machine, and neural net as candidate estimators. Levels of certified cognitive disability ranged from 0 (no cognitive deficits) to 7 (needs constant treatment in a specialized medical facility) according to the severity of their cognitive disability. Thus, larger effect estimates indicate greater level of cognitive disability. The models for home loss exposure were adjusted for the 51 pre-disaster demographic and socioeconomic factors, health conditions, psychosocial variables, and behaviors from the 2010 wave. The models for stroke exposure were adjusted for the 50 pre-disaster factors (i.e., the same adjustment set as the one we used for home loss exposure but excluding baseline stroke).

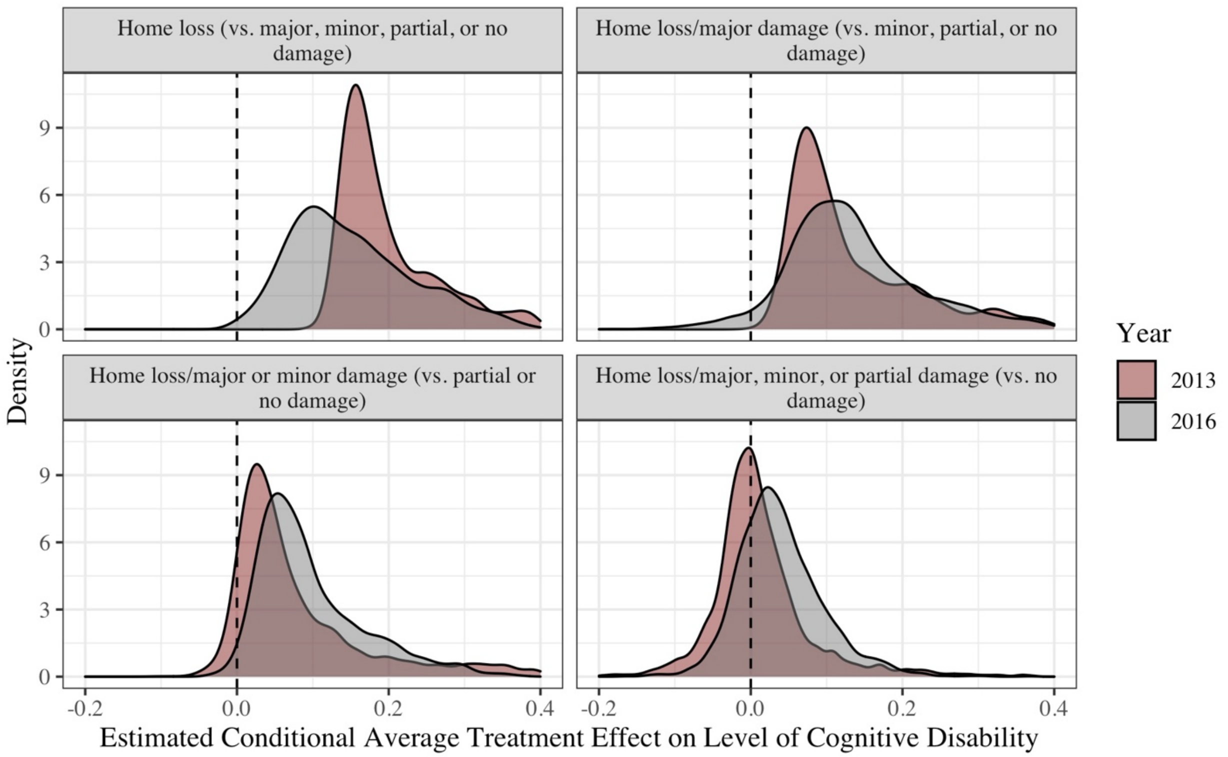

Figure S3. Sensitivity Analysis for Conditional Average Treatment Effects of Housing Damage on Level of Cognitive Disability Living in 2013 and 2016.
Heterogeneous effects (i.e., conditional average treatment effects: CATEs) were estimated using generalized random forest algorithm, using the 51 pre-disaster demographic and socioeconomic factors, health conditions, psychosocial variables, and behaviors from the 2010 wave. Levels of certified cognitive disability ranged from 1 (suffering some cognitive deficits, but otherwise almost completely independent) to 7 (needs constant treatment in a specialized medical facility) according to the severity of their cognitive disability. Thus, larger effect estimates indicate greater level of cognitive disability.

Table S1. Estimates of Population Average Treatment Effects and Conditional Average Treatment Effects of Disaster Damages on Level of Cognitive Disability in 2013 and 2016.

Year	Disaster Exposure	Population Average Effects[a]			Conditional Average Treatment Effects[b]				
		Estimate	(95% CI)	p-value	Mean	SD	Min	Max	P for heterogeneity[c]
2013	Loss of Loved Ones	-0.029	(-0.07, 0.01)	0.19	0.00	0.12	-0.36	1.18	0.78
	Home loss	0.102	(0.07, 0.13)	<0.001	0.25	0.17	0.09	1.56	<0.001
2016	Loss of Loved Ones	-0.003	(-0.06, 0.06)	0.91	0.00	0.07	-0.23	0.49	0.33
	Home loss	0.145	(0.11, 0.18)	<0.001	0.15	0.08	-0.01	0.45	<0.001

[a] Population average effects (i.e., average treatment effects) of the exposures were estimated via the doubly-robust targeted maximum likelihood estimation. Models were estimated data-adaptively via the SuperLearner using generalized linear models, gradient boosting machine, and neural net as candidate estimators. Levels of certified cognitive disability ranged from 0 (no cognitive deficits) to 7 (needs constant treatment in a specialized medical facility) according to the severity of their cognitive disability. Thus, larger effect estimates indicate greater level of cognitive disability. All models were adjusted for the 51 pre-disaster demographic and socioeconomic factors, health conditions, psychosocial variables, and behaviors from the 2010 wave.

[b] Heterogeneous effects (i.e., conditional average treatment effects: CATEs) were estimated using generalized random forest algorithm, using the 51 pre-disaster demographic and socioeconomic factors, health conditions, psychosocial variables, and behaviors from the 2010 wave.

[c] We estimated population average treatment effects among those who were above/below the median CATE. We then computed p-value for the difference between the two estimated average effects as a test for heterogeneity.

Table S2. Pre-disaster Characteristics of People at Top 10% vs Bottom 10% of the Estimated Conditional Average Treatment Effect of Disaster-related Trauma Experiences on Level of Cognitive Disability in 2013 (n = 3,350)

Characteristics	Exposure: Home loss			Exposure: Loss of loved ones		
	Resilient (n = 335)	Vulnerable (n = 335)	p-value	Resilient (n = 335)	Vulnerable (n = 335)	p-value
**CATE Estimates, mean (SD)**	0.14 (0.01)	0.69 (0.22)	<0.001	-0.11 (0.04)	0.27 (0.22)	<0.001
**Age, mean (SD)**	68.97 (2.97)	81.95 (6.54)	<0.001	76.70 (4.92)	79.80 (7.22)	<0.001
**Gender, n (%)**			<0.001			0.13
Men	135 (40%)	202 (60%)		191 (57%)	210 (63%)	
Women	200 (60%)	133 (40%)		144 (43%)	125 (37%)	
**Marital status, n (%)**			<0.001			0.046
Married	314 (94%)	167 (50%)		214 (64%)	182 (54%)	
Widowed	16 (4.8%)	154 (46%)		102 (30%)	139 (41%)	
Divorced	3 (0.9%)	5 (1.5%)		8 (2.4%)	7 (2.1%)	
Single	2 (0.6%)	7 (2.1%)		8 (2.4%)	5 (1.5%)	
Others	0 (0%)	2 (0.6%)		3 (0.9%)	2 (0.6%)	
**Living alone, n (%)**			<0.001			0.2
No	329 (98%)	304 (91%)		299 (89%)	309 (92%)	
Yes	6 (1.8%)	31 (9.3%)		36 (11%)	26 (7.8%)	
**Education, n (%)**			<0.001			<0.001
Less than 6 years	1 (0.3%)	17 (5.1%)		5 (1.5%)	10 (3.0%)	
6-9 years	108 (32%)	192 (57%)		133 (40%)	185 (55%)	
10-12 years	149 (44%)	89 (27%)		130 (39%)	98 (29%)	
13 years or more	72 (21%)	33 (9.9%)		64 (19%)	38 (11%)	
Others	5 (1.5%)	4 (1.2%)		3 (0.9%)	4 (1.2%)	
**Job, n (%)**			<0.001			<0.001
Working	105 (31%)	28 (8.4%)		41 (12%)	31 (9.3%)	
Retired	210 (63%)	180 (54%)		219 (65%)	161 (48%)	
Never worked	20 (6.0%)	127 (38%)		75 (22%)	143 (43%)	
**Household income, mean (SD)**	226.56 (158.95)	211.83 (131.16)	0.4	175.16 (131.90)	213.81 (125.11)	<0.001
**Depressive symptoms, n (%)**			<0.001			0.3
Mild/severe depressive symptoms	65 (19%)	173 (52%)		147 (44%)	161 (48%)	
No depressive symptoms	270 (81%)	162 (48%)		188 (56%)	174 (52%)	
**Self-rated health, n (%)**			<0.001			<0.001
Very good	71 (21%)	20 (6.0%)		35 (10%)	17 (5.1%)	
Good	244 (73%)	184 (55%)		220 (66%)	184 (55%)	
Not good	20 (6.0%)	94 (28%)		66 (20%)	111 (33%)	
Bad	0 (0%)	37 (11%)		14 (4.2%)	23 (6.9%)	
**Body mass index, mean (SD)**	25.26 (2.39)	23.03 (3.30)	<0.001	23.40 (3.47)	23.46 (3.35)	0.14
**Total IADL, mean (SD)**	12.47 (0.80)	8.96 (3.19)	<0.001	12.07 (1.05)	8.57 (3.11)	<0.001
**ADL, mean (SD)**	3.00 (0.00)	2.88 (0.38)	<0.001	2.99 (0.12)	2.91 (0.36)	<0.001
**# of Treatment for major diseases, n (%)**	1.17 (1.08)	2.04 (1.69)	<0.001	1.96 (1.70)	1.90 (1.65)	0.7
**Trust in local people, n (%)**			<0.001			0.15
Very much	18 (5.4%)	60 (18%)		47 (14%)	51 (15%)	
Moderate	209 (62%)	150 (45%)		189 (56%)	157 (47%)	
Neutral	75 (22%)	101 (30%)		80 (24%)	101 (30%)	
Not very much	30 (9.0%)	19 (5.7%)		16 (4.8%)	21 (6.3%)	
Not at all	3 (0.9%)	5 (1.5%)		3 (0.9%)	5 (1.5%)	
**Mutual help in community, n (%)**			<0.001			0.13
Very much	10 (3.0%)	41 (12%)		26 (7.8%)	38 (11%)	
Moderate	171 (51%)	151 (45%)		174 (52%)	150 (45%)	
Neutral	106 (32%)	94 (28%)		103 (31%)	103 (31%)	
Not very much	40 (12%)	34 (10%)		26 (7.8%)	31 (9.3%)	

Not at all	8 (2.4%)	15 (4.5%)		6 (1.8%)	13 (3.9%)	
**Attachment to community, n (%)**			<0.001			0.12
Very much	72 (21%)	107 (32%)		92 (27%)	107 (32%)	
Moderate	188 (56%)	140 (42%)		166 (50%)	142 (42%)	
Neutral	60 (18%)	57 (17%)		62 (19%)	58 (17%)	
Not very much	14 (4.2%)	23 (6.9%)		12 (3.6%)	20 (6.0%)	
Not at all				3 (0.9%)	8 (2.4%)	
**Participation in sport clubs, n (%)**			<0.001			0.021
Everyday	4 (1.2%)	1 (0.3%)		1 (0.3%)	1 (0.3%)	
A few times a week	42 (13%)	7 (2.1%)		13 (3.9%)	18 (5.4%)	
Once a week	34 (10%)	7 (2.1%)		19 (5.7%)	9 (2.7%)	
1-2 times a month	24 (7.2%)	4 (1.2%)		12 (3.6%)	4 (1.2%)	
A few times a year	26 (7.8%)	4 (1.2%)		14 (4.2%)	6 (1.8%)	
Not at all	205 (61%)	312 (93%)		276 (82%)	297 (89%)	
**Participation in hobby clubs, n (%)**			<0.001			0.012
Everyday	7 (2.1%)	1 (0.3%)		2 (0.6%)	2 (0.6%)	
A few times a week	36 (11%)	9 (2.7%)		14 (4.2%)	19 (5.7%)	
Once a week	41 (12%)	11 (3.3%)		28 (8.4%)	15 (4.5%)	
1-2 times a month	67 (20%)	22 (6.6%)		44 (13%)	25 (7.5%)	
A few times a year	44 (13%)	11 (3.3%)		25 (7.5%)	17 (5.1%)	
Not at all	140 (42%)	281 (84%)		222 (66%)	257 (77%)	
**Frequency of meeting friends, n (%)**			<0.001			<0.001
Every day	74 (22%)	35 (10%)		36 (11%)	41 (12%)	
2-3 times a week	81 (24%)	56 (17%)		65 (19%)	63 (19%)	
Once a week	54 (16%)	37 (11%)		60 (18%)	44 (13%)	
1-2 times a month	70 (21%)	54 (16%)		73 (22%)	48 (14%)	
A few times a year	56 (17%)	53 (16%)		79 (24%)	58 (17%)	
Not at all	0 (0%)	100 (30%)		22 (6.6%)	81 (24%)	
**# of friends interacted last month, n (%)**			<0.001			<0.001
0	2 (0.6%)	72 (21%)		12 (3.6%)	71 (21%)	
1-2 friends	20 (6.0%)	108 (32%)		70 (21%)	97 (29%)	
3-5 friends	74 (22%)	87 (26%)		118 (35%)	83 (25%)	
6-9 friends	53 (16%)	23 (6.9%)		38 (11%)	25 (7.5%)	
10 or more friends	186 (56%)	45 (13%)		97 (29%)	59 (18%)	
**Received emotional social support, n (%)**	13 (3.9%)	46 (14%)	<0.001	30 (9.0%)	42 (13%)	0.13
**Provision of emotional support, n (%)**	9 (2.7%)	73 (22%)	<0.001	32 (9.6%)	66 (20%)	<0.001
**Received care support, n (%)**	6 (1.8%)	17 (5.1%)	0.02	19 (5.7%)	17 (5.1%)	0.7
**Provision of care support, n (%)**	18 (5.4%)	112 (33%)	<0.001	46 (14%)	98 (29%)	<0.001
**Communication with neighbors, n (%)**			<0.001			<0.001
Very much	87 (26%)	66 (20%)		109 (33%)	66 (20%)	
Moderate	182 (54%)	158 (47%)		167 (50%)	168 (50%)	
Minimum	63 (19%)	101 (30%)		59 (18%)	89 (27%)	
Not at all	3 (0.9%)	10 (3.0%)		0 (0%)	12 (3.6%)	
**# of negative events in the past year, mean (SD)**	0.55 (0.75)	0.76 (0.83)	<0.001	0.81 (0.80)	0.65 (0.78)	0.002
**Sense of coherence, mean (SD)**	23.08 (3.82)	20.73 (4.15)	<0.001	20.68 (4.09)	21.11 (4.27)	0.1
**Current smoking status, n (%)**			<0.001			0.3
Never	165 (49%)	221 (66%)		204 (61%)	224 (67%)	
Quit	113 (34%)	89 (26.6%)		95 (28.3%)	81 (24.2%)	
Smoking	57 (17%)	25 (7.5%)		36 (11%)	30 (9.0%)	
**Current alcohol drinking, n (%)**			<0.001			0.046
Drinking	184 (55%)	78 (23%)		103 (31%)	81 (24%)	
Quit	13 (3.9%)	12 (3.6%)		18 (5.4%)	11 (3.3%)	
Never	138 (41%)	245 (73%)		214 (64%)	243 (73%)	
**Frequency of fish and meat consumption, n (%)**			0.9			0.11

Twice a day or more often	33 (9.9%)	42 (13%)		32 (9.6%)	33 (9.9%)	
Once a day	119 (36%)	122 (36%)		118 (35%)	120 (36%)	
4-6 times a week	84 (25%)	66 (20%)		84 (25%)	65 (19%)	
2-3 times a week	78 (23%)	81 (24%)		84 (25%)	87 (26%)	
Once a week	14 (4.2%)	15 (4.5%)		13 (3.9%)	18 (5.4%)	
Less than once a week	7 (2.1%)	4 (1.2%)		4 (1.2%)	8 (2.4%)	
Not at all	0 (0%)	5 (1.5%)		0 (0%)	4 (1.2%)	
**Frequency of fruits and vegetable consumption, n (%)**			0.03			0.049
Twice a day or more often	191 (57%)	153 (46%)		165 (49%)	162 (48%)	
Once a day	100 (30%)	118 (35%)		116 (35%)	102 (30%)	
4-6 times a week	26 (7.8%)	28 (8.4%)		36 (11%)	29 (8.7%)	
2-3 times a week	14 (4.2%)	27 (8.1%)		15 (4.5%)	30 (9.0%)	
Once a week	2 (0.6%)	5 (1.5%)		2 (0.6%)	6 (1.8%)	
Less than once a week	2 (0.6%)	2 (0.6%)		1 (0.3%)	4 (1.2%)	
Not at all	0 (0%)	2 (0.6%)		0 (0%)	2 (0.6%)	
**Frequency of going out, n (%)**			<0.001			<0.001
Every day	225 (67%)	62 (19%)		148 (44%)	71 (21%)	
2-3 times a week	85 (25%)	89 (27%)		126 (38%)	85 (25%)	
Once a week	18 (5.4%)	67 (20%)		48 (14%)	54 (16%)	
1-2 times a month	7 (2.1%)	54 (16%)		10 (3.0%)	68 (20%)	
A few times a year	0 (0%)	20 (6.0%)		2 (0.6%)	23 (6.9%)	
Not at all	0 (0%)	43 (13%)		1 (0.3%)	34 (10%)	
**Having hobby, n (%)**			<0.001			<0.001
Yes	234 (70%)	106 (32%)		169 (50%)	122 (36%)	
No	101 (30%)	229 (68%)		166 (50%)	213 (64%)	

Table S3. Pre-disaster Characteristics of People at Bottom 10% vs Top 10% of the Estimated Conditional Average Treatment Effect of Disaster-related Trauma Experiences on Level of Cognitive Disability in 2016 (n = 2,664)

Characteristics	Exposure: Home loss			Exposure: Loss of loved ones		
	Resilient (n = 267)	Vulnerable (n = 267)	p-value	Resilient (n = 267)	Vulnerable (n = 267)	p-value
**CATE Estimates, mean (SD)**[c]	0.03 (0.02)	0.34 (0.04)	<0.001	-0.11 (0.03)	0.16 (0.07)	<0.001
**Age, mean (SD)**	68.55 (2.66)	80.45 (3.15)	<0.001	78.79 (5.16)	74.17 (6.00)	<0.001
**Gender, n (%)**			0.027			0.1
Men	172 (64%)	147 (55%)		145 (54%)	164 (61%)	
Women	95 (36%)	120 (45%)		122 (46%)	103 (39%)	
**Marital status, n (%)**			<0.001			0.03
Married	221 (83%)	171 (64%)		192 (72%)	165 (62%)	
Widowed	33 (12%)	83 (31%)		67 (25%)	83 (31%)	
Divorced	9 (3.4%)	6 (2.2%)		4 (1.5%)	10 (3.7%)	
Single	2 (0.7%)	6 (2.2%)		4 (1.5%)	5 (1.9%)	
Others	2 (0.7%)	1 (0.4%)		0 (0%)	4 (1.5%)	
**Living alone, n (%)**			0.093			<0.001
No	249 (93%)	238 (89%)		254 (95%)	230 (86%)	
Yes	18 (6.7%)	29 (11%)		13 (4.9%)	37 (14%)	
**Education, n (%)**			0.004			<0.001
Less than 6 years	1 (0.4%)	5 (1.9%)		3 (1.1%)	6 (2.2%)	
6-9 years	72 (27%)	95 (36%)		92 (34%)	138 (52%)	
10-12 years	145 (54%)	105 (39%)		106 (40%)	90 (34%)	
13 years or more	48 (18%)	59 (22%)		63 (24%)	29 (11%)	
Others	1 (0.4%)	3 (1.1%)		3 (1.1%)	4 (1.5%)	
**Job, n (%)**			<0.001			0.008
Working	68 (25%)	15 (5.6%)		30 (11%)	33 (12%)	
Retired	156 (58%)	198 (74%)		186 (70%)	154 (58%)	
Never worked	43 (16%)	54 (20%)		51 (19%)	80 (30%)	
**Household income, mean (SD)**	186.98 (143.62)	275.50 (115.46)	<0.001	219.90 (142.92)	180.80 (108.11)	<0.001
**Depressive symptoms, n (%)**			<0.001			<0.001
Mild/severe depressive symptoms	34 (13%)	81 (30%)		22 (8.2%)	206 (77%)	
No depressive symptoms	233 (87%)	186 (70%)		245 (92%)	61 (23%)	
**Self-rated health, n (%)**			<0.001			<0.001
Very good	38 (14%)	27 (10%)		39 (15%)	7 (2.6%)	
Good	211 (79%)	191 (72%)		203 (76%)	134 (50%)	
Not good	17 (6.4%)	40 (15%)		23 (8.6%)	104 (39%)	
Bad	1 (0.4%)	9 (3.4%)		2 (0.7%)	22 (8.2%)	
**Body mass index, mean (SD)**	24.69 (3.47)	22.80 (2.46)	<0.001	22.39 (2.50)	23.76 (3.45)	<0.001
**Total IADL, mean (SD)**	12.03 (1.46)	12.17 (1.50)	0.053	12.38 (0.81)	9.23 (2.58)	<0.001
**ADL, mean (SD)**	3.00 (0.00)	3.00 (0.06)	0.3	3.00 (0.00)	2.96 (0.23)	0.004
**# of Treatment for major diseases, n (%)**	1.21 (1.14)	1.77 (1.51)	<0.001	1.62 (1.40)	1.75 (1.63)	0.6
**Trust in local people, n (%)**			0.11			<0.001
Very much	25 (9.4%)	35 (13%)		53 (20%)	25 (9.4%)	
Moderate	162 (61%)	152 (57%)		176 (66%)	98 (37%)	
Neutral	63 (24%)	68 (25%)		33 (12%)	109 (41%)	
Not very much	17 (6.4%)	9 (3.4%)		5 (1.9%)	30 (11%)	
Not at all	0 (0%)	3 (1.1%)		0 (0%)	5 (1.9%)	
**Mutual help in community, n (%)**			0.009			<0.001
Very much	12 (4.5%)	32 (12%)		36 (13%)	12 (4.5%)	
Moderate	145 (54%)	135 (51%)		162 (61%)	97 (36%)	
Neutral	86 (32%)	84 (31%)		58 (22%)	111 (42%)	
Not very much	21 (7.9%)	11 (4.1%)		11 (4.1%)	34 (13%)	

Not at all	3 (1.1%)	5 (1.9%)		0 (0%)	13 (4.9%)	
**Attachment to community, n (%)**			<0.001			<0.001
Very much	46 (17%)	88 (33%)		112 (42%)	42 (16%)	
Moderate	160 (60%)	139 (52%)		128 (48%)	115 (43%)	
Neutral	48 (18%)	26 (9.7%)		19 (7.1%)	66 (25%)	
Not very much	10 (3.7%)	12 (4.5%)		8 (3.0%)	37 (14%)	
Not at all				0 (0%)	7 (2.6%)	
**Participation in sport clubs, n (%)**			<0.001			<0.001
Everyday	1 (0.4%)	2 (0.7%)		2 (0.7%)	0 (0%)	
A few times a week	11 (4.1%)	41 (15%)		35 (13%)	5 (1.9%)	
Once a week	13 (4.9%)	35 (13%)		25 (9.4%)	4 (1.5%)	
1-2 times a month	9 (3.4%)	14 (5.2%)		17 (6.4%)	3 (1.1%)	
A few times a year	12 (4.5%)	13 (4.9%)		19 (7.1%)	7 (2.6%)	
Not at all	221 (83%)	162 (61%)		169 (63%)	248 (93%)	
**Participation in hobby clubs, n (%)**			<0.001			<0.001
Everyday	0 (0%)	2 (0.7%)		1 (0.4%)	1 (0.4%)	
A few times a week	8 (3.0%)	43 (16%)		32 (12%)	6 (2.2%)	
Once a week	13 (4.9%)	44 (16%)		38 (14%)	18 (6.7%)	
1-2 times a month	24 (9.0%)	70 (26%)		51 (19%)	17 (6.4%)	
A few times a year	21 (7.9%)	25 (9.4%)		30 (11%)	17 (6.4%)	
Not at all	201 (75%)	83 (31%)		115 (43%)	208 (78%)	
**Frequency of meeting friends, n (%)**			<0.001			<0.001
Every day	3 (1.1%)	59 (22%)		33 (12%)	15 (5.6%)	
2-3 times a week	38 (14%)	61 (23%)		80 (30%)	39 (15%)	
Once a week	47 (18%)	53 (20%)		56 (21%)	52 (19%)	
1-2 times a month	73 (27%)	54 (20%)		58 (22%)	48 (18%)	
A few times a year	75 (28%)	33 (12%)		34 (13%)	60 (22%)	
Not at all	31 (12%)	7 (2.6%)		6 (2.2%)	53 (20%)	
**# of friends interacted last month, n (%)**			<0.001			<0.001
0	24 (9.0%)	12 (4.5%)		7 (2.6%)	39 (15%)	
1-2 friends	58 (22%)	26 (9.7%)		33 (12%)	86 (32%)	
3-5 friends	101 (38%)	76 (28%)		82 (31%)	72 (27%)	
6-9 friends	25 (9.4%)	42 (16%)		32 (12%)	25 (9.4%)	
10 or more friends	59 (22%)	111 (42%)		113 (42%)	45 (17%)	
**Received emotional social support, n (%)**	14 (5.2%)	9 (3.4%)	0.3	4 (1.5%)	34 (13%)	<0.001
**Provision of emotional support, n (%)**	17 (6.4%)	16 (6.0%)	0.9	7 (2.6%)	51 (19%)	<0.001
**Received care support, n (%)**	8 (3.0%)	6 (2.2%)	0.6	2 (0.7%)	27 (10%)	<0.001
**Provision of care support, n (%)**	19 (7.1%)	32 (12%)	0.056	21 (7.9%)	69 (26%)	<0.001
**Communication with neighbors, n (%)**			<0.001			<0.001
Very much	26 (9.7%)	84 (31%)		82 (31%)	36 (13%)	
Moderate	178 (67%)	143 (54%)		159 (60%)	130 (49%)	
Minimum	61 (23%)	39 (15%)		26 (9.7%)	93 (35%)	
Not at all	2 (0.7%)	1 (0.4%)		0 (0%)	8 (3.0%)	
**# of negative events in the past year, mean (SD)**	0.48 (0.66)	0.71 (0.73)	<0.001	0.65 (0.77)	0.82 (0.85)	0.021
**Sense of coherence, mean (SD)**	23.79 (3.02)	21.76 (4.19)	<0.001	24.46 (2.82)	18.19 (3.93)	<0.001
**Current smoking status, n (%)**			0.4			0.035
Never	192 (72%)	176 (66%)		178 (67%)	175 (66%)	
Quit	57 (22%)	73 (27%)		68 (26%)	60 (23%)	
Smoking	18 (6.7%)	18 (6.7%)		21 (7.9%)	32 (12%)	
**Current alcohol drinking, n (%)**			0.039			0.4
Drinking	107 (40%)	80 (30%)		89 (33%)	84 (31%)	
Quit	6 (2.2%)	10 (3.7%)		11 (4.1%)	6 (2.2%)	
Never	154 (58%)	177 (66%)		167 (63%)	177 (66%)	
**Frequency of fish and meat consumption, n (%)**			0.049			<0.001

Twice a day or more often	28 (10%)	39 (15%)		50 (19%)	18 (6.7%)	
Once a day	118 (44%)	108 (40%)		111 (42%)	74 (28%)	
4-6 times a week	67 (25%)	48 (18%)		39 (15%)	55 (21%)	
2-3 times a week	42 (16%)	64 (24%)		61 (23%)	88 (33%)	
Once a week	8 (3.0%)	6 (2.2%)		4 (1.5%)	25 (9.4%)	
Less than once a week	4 (1.5%)	2 (0.7%)		2 (0.7%)	6 (2.2%)	
Not at all	0 (0%)	0 (0%)		0 (0%)	1 (0.4%)	
**Frequency of fruits and vegetable consumption, n (%)**			0.002			<0.001
Twice a day or more often	173 (65%)	131 (49%)		163 (61%)	100 (37%)	
Once a day	68 (25%)	85 (32%)		80 (30%)	103 (39%)	
4-6 times a week	15 (5.6%)	30 (11%)		18 (6.7%)	28 (10%)	
2-3 times a week	10 (3.7%)	18 (6.7%)		5 (1.9%)	29 (11%)	
Once a week	1 (0.4%)	3 (1.1%)		1 (0.4%)	4 (1.5%)	
Less than once a week	0 (0%)	0 (0%)		0 (0%)	3 (1.1%)	
Not at all	0 (0%)	0 (0%)		0 (0%)	0 (0%)	
**Frequency of going out, n (%)**			0.049			<0.001
Every day	127 (48%)	147 (55%)		144 (54%)	57 (21%)	
2-3 times a week	98 (37%)	98 (37%)		88 (33%)	69 (26%)	
Once a week	26 (9.7%)	11 (4.1%)		30 (11%)	53 (20%)	
1-2 times a month	10 (3.7%)	8 (3.0%)		2 (0.7%)	54 (20%)	
A few times a year	3 (1.1%)	3 (1.1%)		1 (0.4%)	20 (7.5%)	
Not at all	3 (1.1%)	0 (0%)		2 (0.7%)	14 (5.2%)	
**Having hobby, n (%)**			<0.001			<0.001
Yes	240 (90%)	96 (36%)		184 (69%)	83 (31%)	
No	27 (10%)	171 (64%)		83 (31%)	184 (69%)	

Table S4. Criteria for Levels of Cognitive Disability in the Japanese Long-term Care Insurance System.

Disability Rank	Outcome value	Criteria	Examples of observable symptoms or behaviors
	0	No cognitive deficit.	
I	1	Suffers from some cognitive disability, but the daily living is almost all independent in the domestic and social spheres.	
II		Manifests some symptoms/behaviors and communication difficulties that may hinder the daily activities but can be independent if someone takes care of them.	
IIa	2	The abovementioned conditions in II are observed while outside the domestic sphere.	Frequently gets lost on the street or makes noticeable mistakes in matters that the person was previously able to handle, such as shopping, personal administrative tasks, or financial management.
IIb	3	The abovementioned conditions in II are also observed within the domestic sphere.	Is unable to manage taking medication or staying alone at home due to an inability to answer the phone or the door.
III		Occasionally manifests communication difficulties or symptoms/behaviors that hinder daily activities, thus requiring care.	
IIIa	4	Manifests abovementioned conditions described in III predominantly during the day.	Has difficulty or takes time to change clothes, take meals, defecate, or urinate; puts objects into the mouth, picks up and collects objects, is incontinent, makes loud and incoherent screams, carelessly handles fire, or engages in unhygienic acts or inappropriate sexual acts, etc.
IIIb	5	Manifests abovementioned conditions described in III predominantly at night.	Same as rank IIIa.
IV	6	Frequently manifests difficulties in communicating or symptoms/behaviors that hinder daily activities and constantly requires care.	Same as rank III.
M	7	Manifests significant mental symptoms, problematic behaviors, orsevere physical illnesses and requires specialized medical care.	Shows continued mental symptoms, such as delirium, delusions, andagitation, and manifests associated problematic behaviors, such asself-mutilation or harm to others.

Table S5. Distributions of Levels of Certified Cognitive Disability in 2013 and 2016 Among the Analytic Samples (n = 3,350 for 2013 and n = 2,264 for 2016).

Characteristics	Overall	Home loss		Loss of loved ones	
		Yes	No	Yes	No
	n (%)	n (%)	n (%)	n (%)	n (%)
**Levels of certified cognitive disability in 2013**[b]					
Total	3,350 (100)	148 (100)	3,112 (100)	1,254 (100)	2,096 (100)
0 (no cognitive deficits)	3,064 (91%)	122 (82%)	2,865 (92%)	1,153 (92%)	1,911 (91%)
1	50 (1.5%)	2 (1.4%)	47 (1.5%)	22 (1.8%)	28 (1.3%)
2	104 (3.1%)	8 (5.4%)	88 (2.8%)	38 (3.0%)	66 (3.1%)
3	33 (1.0%)	4 (2.7%)	29 (0.9%)	6 (0.5%)	27 (1.3%)
4	67 (2.0%)	9 (6.1%)	55 (1.8%)	25 (2.0%)	42 (2.0%)
5	20 (0.6%)	1 (0.7%)	19 (0.6%)	7 (0.6%)	13 (0.6%)
6	9 (0.3%)	0 (0%)	8 (0.3%)	1 (<0.1%)	8 (0.4%)
7 (needs constant treatment in a specialized medical facility)	3 (<0.1%)	2 (1.4%)	1 (<0.1%)	2 (0.2%)	1 (<0.1%)
**Levels of certified cognitive disability in 2016**[b]					
Total	2,264 (100)	107 (100)	2,496 (100)	1,041 (100)	1,623 (100)
0 (no cognitive deficits)	2,308 (87%)	85 (79%)	2,171 (87%)	904 (87%)	1,404 (87%)
1	39 (1.5%)	1 (0.9%)	37 (1.5%)	17 (1.6%)	22 (1.4%)
2	173 (6.5%)	12 (11%)	156 (6.2%)	71 (6.8%)	102 (6.3%)
3	37 (1.4%)	2 (1.9%)	35 (1.4%)	13 (1.2%)	24 (1.5%)
4	69 (2.6%)	6 (5.6%)	61 (2.4%)	23 (2.2%)	46 (2.8%)
5	28 (1.1%)	1 (0.9%)	26 (1.0%)	10 (1.0%)	18 (1.1%)
6	9 (0.3%)	0 (0%)	9 (0.4%)	3 (0.3%)	6 (0.4%)
7 (needs constant treatment in a specialized medical facility)	1 (<0.1%)	0 (0%)	1 (<0.1%)	0 (0%)	1 (<0.1%)

Table S6. Levels of Housing Damage and Criterion Certifying by Local Governments

Grade	Criterion [a]
No damage	Not affected.
Partial	Under 20% structural damage or inundation below the floor.
Minor	20% to 40% structural damage or inundation above the floor.
Major	40% to 50% structural damage or inundation approximately 1 meter above the floor.
Complete destruction (Home loss)	Over 50% structural damage, inundation up to ceiling in the first floor, or completely washed away. Uninhabitable beyond repair.

[a] Structural damage was observed in roof, walls, and foundation.

Table S7. List of Baseline Covariates

Variable Type	Measurement	Coding
Demographic	Gender	1= Man; 2 = Women
Demographic	Age	As continuous
Demographic	Marital status	1 = Married; 2 = Widowed; 3 = Divorced; 4 = Single; 5 = Others
Demographic	Living alone	0 = No; 1 = Yes
Socioeconomic	Education attainment	1= Less than 6 years; 2 = 6-9 years; 3 = 10-12 years; 4 = 13-15 years; 5 = Others
Socioeconomic	Employment status	1= Working; 2 = Retired; 3 = Never worked
Socioeconomic	Equivalised household income	As continuous
Health	Self-rated health	1= Very good; 2 = Good; 3 = Not good; 4 = Bad
Health	Body Mass Index	As continuous
Health	Depressive Symptoms	As continuous
Health	ADL	1= Completely needed; 2 = Partially needed; 3 = No help needed
Health	IADL	As continuous
Health	All self-reported diagnosis of 19 major diseases (cancer, heart diseases, stroke, hypertension, diabetes, obesity, hyperlipidemia, osteoporosis, arthritis, fracture, respiratory diseases, gastrointestinal diseases, liver diseases, psychiatric diseases, dysphagia, visual impairment, hearing loss, dysuria, and insomnia)	0 = No; 1 = Yes for each disease.
Psychosocial	Trust in local people (Can you trust your local people?)	1 = Very much; 2 = Moderate; 3 = Neutral; 4 = Not very much; 5 = Not at all
Psychosocial	Mutual help in your community	1 = Very much; 2 = Moderate; 3 = Neutral; 4 = Not very much; 5 = Not at all
Psychosocial	How much do you attach your community?	1 = Very much; 2 = Moderate; 3 = Neutral; 4 = Not very much; 5 = Not at all
Psychosocial	Participation in sport clubs	1 = Everyday; 2 = 2-3times a week; 3 = once a week; 4 = 1-2 times a month; 5 = a few times a year; 6 = not at all
Psychosocial	Participation in hobby clubs	1 = Everyday; 2 = 2-3 times a week; 3 = once a week; 4 = 1-2 times a month; 5 = a few times a year; 6 = not at all
Psychosocial	Meeting friends	1 = Every day; 2 =2-3 times a year; 3 = once a week; 4 = 1-2 times a month; 5 = a few times a year, 6 = Not at all
Psychosocial	How many friends did you meet in the past month?	1 = 0; 2 = 1-2 friends; 3 = 3-5 friends; 4 = 6-9 friends; 5 = 10 or more friends
Psychosocial	Received emotional support	0 = Yes; 1 = No
Psychosocial	Providing emotional support	0 = Yes; 1 = No
Psychosocial	Received care support	0 = Yes; 1 = No
Psychosocial	Giving care support	0 = Yes; 1 = No
Psychosocial	Communication with neighbors	1 = Very much; 2 = Moderate; 3 = Minimum; 4 = Not at all
Psychosocial	Count of 6 negative life events	As continuous
Psychosocial	Sense of coherence	As continuous
Behavioral	Current smoking status	1 = Never; 2 = Quit; 3 = Smoking
Behavioral	Current alcohol drinking	1 = Drinking; 2 = Quit; 3 = No drinking
Behavioral	Frequency of fish and meat consumption	1 = Twice a day or more often; 2 = once a day; 3 = 4-6 times a week; 4 = 2-3 times a week; 5 = once a week; 6 = less than once a week; 7 = not at all

Behavioral	Eating vegetable or fruit	1 = Twice a day or more often; 2 = once a day; 3 = 4-6 times a week; 4 = 2-3 times a week; 5 = once a week; 6 = less than once a week; 7 = not at all
Behavioral	Frequency of going out	1 = Everyday; 2 = 2-3 times a week; 3 = once a week; 4 = 1-2 times a month; 5 = a few times a year; 6 = not at all
Behavioral	Having hobby	1 = Yes; 2 = No

All covariates were measured at the 2010 wave, 7 months prior to the disaster onset. These variables were used as covariates to control for confounding and also to estimate effect heterogeneity.
